# Effect of Aging on Muscle-Pump Baroreflex of Individual Leg Muscles During Standing

**DOI:** 10.3389/fphys.2019.00845

**Published:** 2019-07-16

**Authors:** Ajay K. Verma, Da Xu, Amanmeet Garg, Andrew P. Blaber, Kouhyar Tavakolian

**Affiliations:** ^1^School of Electrical Engineering and Computer Science, University of North Dakota, Grand Forks, ND, United States; ^2^Department of Biomedical Physiology and Kinesiology, Simon Fraser University, Burnaby, BC, Canada

**Keywords:** orthostatic intolerance, falls, cardiac baroreflex, blood pressure regulation, cardio-postural control

## Abstract

Activation of leg muscles is an important component in the regulation of blood pressure during standing, failure of which could result in syncope and falls. Our previous work demonstrated baroreflex mediated activation of leg muscles (muscle-pump baroreflex) as an important factor in the regulation of blood pressure during standing; however, the effect of aging on the muscle-pump baroreflex of individual leg muscles during standing remains to be understood. Here, the interaction between systolic blood pressure (SBP) and the activation of lateral gastrocnemius (LG), medial gastrocnemius (MG), tibialis anterior (TA), and soleus (SOL) muscles during standing was quantified. Beat-to-beat heart period (RR interval), SBP, electromyography impulse (EMG_imp_) were derived from continuously acquired electrocardiography, finger blood pressure, and calf-electromyography, respectively. The cardiac baroreflex (SBP→RR) causality (0.88 ± 0.08 vs. 0.94 ± 0.03, *p* = 0.01), percent time with significant coherence (%SC: 50.95 ± 23.31 vs. 76.75 ± 16.91, *p* = 0.001), and gain (4.39 ± 4.38 vs. 13.05 ± 8.11, *p* < 0.001) was lower in older (69 ± 4 years) compared to young (26 ± 2 years) persons. Muscle-pump baroreflex (SBP→EMG_imp_) causality of LG (0.81 ± 0.08 vs. 0.88 ± 0.05, *p* = 0.01) and SOL (0.79 ± 0.11 vs. 0.88 ± 0.04, *p* = 0.01) muscles was lower in older compared to young persons. %SC was lower for all muscles in the older group (LG, *p* < 0.001; MG, *p* = 0.01; TA, *p* = 0.01; and SOL, *p* < 0.001) compared to young. The study outcomes highlighted impairment in muscle-pump baroreflex with age in addition to cardiac baroreflex. The findings of the study can assist in the development of an effective system for monitoring orthostatic tolerance via cardiac and muscle-pump baroreflexes to mitigate syncope and falls.

## Introduction

Falls are debilitating events which occur with increasing frequency with age and cause injuries needing urgent medical attention, degradation in the quality of life, as well as significant financial burden on the healthcare system (Gazibara et al., 2014; Terroso et al., 2014; Do et al., 2015). Injuries associated with non-fatal falls accounted for over 31 billion dollars in the United States (Burns et al., 2016). With older persons expected to double in the United States by the year 2050 compared to 2012, increase in fall-related incidents are anticipated (Ortman et al., 2014). Orthostatic hypotension is one of the primary causes of falls, affecting older persons, and even more adversely, people with neurological disorders (Allcock et al., 2004; Shaw and Claydon, 2014; Shaw et al., 2015). Therefore, technological development for assessment of orthostatic tolerance can play a vital role in mitigating such incidents and improving quality of life.

Standing induces displacement of blood volume footward, therefore challenging blood pressure homeostasis (Rowell, 1993). In young healthy people, blood pressure is regulated during upright stance via a series of reflex mechanisms, of which baroreflex is a key component (Heesch, 1999; Verma et al., 2018). However, in older persons, due to a decline in the sensitivity of baroreceptor response and the inability to increase vascular resistance via sympathetic outflow, regulating blood pressure can be challenging during standing (Monahan, 2007). Accordingly, orthostatic hypotension (OH), a physiological condition where blood pressure drops abruptly (i.e., 20 mmHg in systolic blood pressure or 10 mmHg for diastolic blood pressure) within 3-min of assuming upright stance, is frequently experienced by older persons (Senard et al., 1997; Eigenbrodt et al., 2000; Blaber et al., 2013b; Ricci et al., 2015).

Aging not only deteriorates autonomic but also skeletal muscle function (Lynch et al., 1999; Vandervoort, 2002). During standing, in addition to autonomic control of blood pressure (elevation in heart rate and systemic vascular resistance), the lower leg muscles also play a consequential role in the maintenance of blood pressure by propelling the pooled venous blood back to the heart (Rowell, 1993; Halliwill et al., 2014). Therefore, regulation of blood pressure during standing is an integrative process and require continuous input from both cardiovascular and postural systems. The literature typically stresses upon autonomic control of blood pressure when addressing the physiology of orthostatic hypotension (and associated falls). The postural control of blood pressure is often neglected and requires special consideration when accounting for OH. The ability to continuously monitor the interplay between cardiovascular and postural systems during standing could assist early identification of fall proneness, which can facilitate the design of pertinent preventive measures to mitigate unexpected falls and associated injuries which significantly affect mobility.

In this regard, our previous work quantified the physiological interplay between the representative signals of cardiovascular and postural systems during orthostatic challenge (Blaber et al., 2009; Garg et al., 2014b; Verma et al., 2017a; Xu et al., 2017). In addition to the known knowledge of mechanical muscle pump to propel the venous blood back to the heart, the activation of leg muscles was shown to be dependent on blood pressure fluctuations, i.e., muscle-pump baroreflex (Xu et al., 2017). However, the muscle-pump baroreflex response for individual leg muscles in response to orthostatic challenge is yet to be comprehensively investigated (Garg et al., 2014a). Such knowledge can facilitate the design of appropriate exercise and rehabilitation schemes to ascertain the functionality of important blood pressure regulatory muscles to mitigate the adverse effects of aging and neurological disorders.

In the current work, utilizing data from young, healthy individuals, we investigated the role of muscle-pump baroreflex of individual leg muscles namely the lateral gastrocnemius (LG), medial gastrocnemius (MG), tibialis anterior (TA), and soleus (SOL) toward facilitating venous return to the heart. Further, we studied the effect of aging and sex on cardiac and muscle-pump baroreflexes.

The cardiac and muscle-pump baroreflexes were studied via convergent cross mapping (CCM) to gain insights regarding the directionality of the interaction (Sugihara et al., 2012; Schiecke et al., 2016; Verma et al., 2017a,b), while wavelet transform coherence was utilized to study the interdependency between the respective signals of the two systems (Garg et al., 2014b; Xu et al., 2017). The study outcome is expected to unravel the influence of aging on muscle-pump baroreflex of respective leg muscles, a pivotal mechanism which assures stable upright stance via continuous muscle activation in response to gravity-induced blood pooling to regulate blood pressure during standing.

## Materials and Methods

### Experimental Protocol

The experimental design was approved to be of minimal risk by the research ethics board of Simon Fraser University (SFU). Written and informed consent for participation was obtained from all participants prior to experimentation. Participants were screened from the experimentation if they had cardiovascular, respiratory, or neurological diseases and major musculoskeletal diseases. Additionally, participants were required to report the use of medications. Participants consuming any substance, which could potentially alter cardiovascular and/or postural stability, were excluded from experimentation.

The signals were acquired during a 10-min sit-to-stand test. The experimentation protocol required participants to be seated for 5 min and then passively assisted to standing phase to maintain a quiet stance for an additional 5 min. The procedure was conducted blindfolded in a sensory reduced environment. All experimentation was performed at the Aerospace Physiology Laboratory in the Department of Biomedical Physiology and Kinesiology, SFU. The experimentation was terminated immediately if the participant showed signs of discomfort, uneasiness, nausea, or upon request. The experimentation protocols complied with the rules and regulation set forth by the research ethics board of SFU.

### Data Acquisition

Simultaneous electrocardiogram (ECG) in lead II configuration from Lifepak 8 (Medtronic Inc., MN, United States), continuous non-invasive blood pressure using finger photoplethysmography cuff (Finapres, FMS, Netherlands), and calf electromyography (EMG) were obtained from 18 young, healthy (age: 26 ± 2 years, height: 174 ± 8 cm, weight: 68 ± 11 Kg, 8 females) and 14 older (age: 69 ± 4 years, height: 165 ± 13 cm, weight: 66 ± 17 Kg, 8 females) participants. Members of the older group were physically active and were members of a local running club. The EMG from four different leg muscles [tibialis anterior (TA), lateral gastrocnemius (LG), medial gastrocnemius (MG), and soleus (SOL)] using transdermal differential recording was acquired and then rectified. All signal acquisition was with a national instruments data acquisition system (National Instruments Inc., TX, United States) at a sampling rate of 1000 Hz.

### Data Processing

The last 4 min of standing data were analyzed. The QRS complex in an ECG signal was detected using the Pan-Tompkins algorithm (Pan and Tompkins, 1985), from which, R-R interval time series was obtained. Beat-by-beat SBP and DBP were obtained as a maximum and a minimum value in the blood pressure waveform, respectively, between adjacent QRS complexes. Beat-by-beat EMG (EMG impulse, EMG_imp_) for individual muscles was obtained as the mean area under the rectified EMG envelope between adjacent QRS complexes. Details of the EMG preprocessing can be obtained in our previous work (Xu et al., 2017). Aggregate beat-by-beat EMG_imp_ for respective muscle was obtained by adding the EMG_imp_ of the left and right leg.

Beat-by-beat physiological signals were interpolated to create an evenly sampled continuous signals and were resampled to 10 Hz before further processing. The continuous signals were fed into a convergent cross mapping (CCM) algorithm to gain information regarding causal behavior of cardio-postural interaction (Sugihara et al., 2012). The causality values vary between 0 and 1 and signify the strength in feedforward and feedback direction. Prior to causality analysis, appropriate parameters to perform non-linear state space reconstruction of signals under consideration was determined according to a false nearest neighbor algorithm of the CRP toolbox in MATLAB (MathWorks Inc., MA, United States) (Marwan, 2017). The steps to obtain causal relationship between heart period and blood pressure, as well as skeletal muscle activation and blood pressure, are summarized in our previous work (Verma et al., 2017b; Xu et al., 2017).

The Wavelet Transform Coherence (WTC) method proposed by Torrence and Compo (28) was utilized to compute time-frequency distribution using the Morlet Wavelet for SBP→RR and SBP→EMG_imp_ signal pairs (Torrence and Compo, 1998). The threshold for significant coherence was obtained via Monte Carlo simulation using 500 pairs of surrogate data with the 90th percentile of coherence sampling distribution at each scale/frequency. The percentage time of WTC with significant coherence (%SC, varies between 0 and 100) and gain value in very-low-frequency (VLF, 0.03–0.07 Hz), low-frequency (LF, 0.07–0.15 Hz), and high-frequency (HF, 0.15–0.5 Hz) bands were calculated. The WTC analysis was performed as described in our previous work (Xu et al., 2017).

### Statistical Analysis

Test of normality of the study variable means was conducted using the Shapiro–Wilk test (IBM SPSS Statistics, IBM Corporation, Armonk, NY, United States). For normally distributed data one-way ANOVA was employed for multiple comparison, for non-normal distribution the Kruskal–Wallis test was employed for multiple comparison. Effect of aging on the study variables was tested using unpaired *t*-test (for normally distributed data) or Wilcoxon rank sum test (for non-normally distributed data). Whenever necessary, *post hoc* analysis was conducted using Tukey-HSD. The test results were considered significant at α = 0.05. The results are reported as mean ± SD unless noted otherwise. The statistical tests were performed using MATLAB (MathWorks Inc., Natick, MA, United States).

## Results

Given the limited sample size of our study, the distribution of study variable means showed mixed behavior, i.e., the distribution of means were not always normal, therefore Kruskal–Wallis or Wilcoxon rank sum tests were used. No difference in heart period (*p* = 0.92) and diastolic blood pressure (*p* = 0.86) was observed in the older group compared to young. Systolic blood pressure was higher (*p* < 0.001) in the older group compared to young. EMG_imp_ for LG (*p* = 0.07), MG (*p* = 0.09), SOL (*p* = 0.58) muscles were not different in older persons, but it was higher for TA (*p* = 0.001) muscle compared to young (Table 1).

**TABLE 1:** Cardiovascular and postural parameters (mean ± SD) for young and older groups.

**Parameters**	**Young**	**Older**	***p*-Value**
RR (ms)	787.49 ± 131.98	808.73 ± 180.49	0.92
SBP (mmHg)	106.91 ± 9.66	142.10 ± 20.19	<0.001^†^
DBP (mmHg)	67.39 ± 5.89	68.53 ± 9.83	0.86
EMG_imp(LG)_ (μV⋅s)	8.11 ± 6.40	9.18 ± 3.54	0.07
EMG_imp(MG)_ (μV⋅s)	10.66 ± 6.36	14.01 ± 6.87	0.09
EMG_imp(TA)_ (μV⋅s)	7.77 ± 6.92	14.11 ± 8.90	0.001^†^
EMG_imp(SOL)_ (μV⋅s)	13.90 ± 6.89	16.19 ± 6.85	0.58

*Table also lists Wilcoxon rank sum test comparison *p*-value. ^†^Represents significant difference between the two groups.*

In the young group, muscle-pump baroreflex (SBP→EMG_imp_) of MG was significantly lower (Table 2) compared to TA (*p* = 0.02), while no difference (*p* > 0.10) in the strength of feedforward muscle pump (EMG_imp_→SBP) causality was observed among the four muscle groups (Table 2). The SBP→EMG_imp_ %SC in the LF region for MG was lower (*p* = 0.006) compared to LG (Table 2). Muscle-pump baroreflex gain in the LF region was higher for MG (*p* < 0.001) and SOL (*p* = 0.01) compared to LG (Table 2).

**TABLE 2:** Comparative behavior of muscle-pump baroreflex and muscle-pump for different leg muscles in young group.

**Muscles for comparison**	**LG vs. MG**	**LG vs. TA**	**LG vs. SOL**	**MG vs. TA**	**MG vs. SOL**	**TA vs. SOL**
SBP→EMG_imp_ causality	0.10	0.98	0.97	0.04	0.26	0.87
EMG_imp_→SBP causality	0.94	0.99	0.99	0.99	0.84	0.94
SBP→EMG_imp_ %SC	0.006	0.24	0.73	0.52	0.12	0.83
SBP→EMG_imp_ gain	<0.001	0.75	0.01	0.02	0.87	0.17

*The table list the multiple comparison (Kruskal–Wallis test followed by Tukey-HSD) *p*-value. Results were considered significant at *p* ≤ 0.05.*

**FIGURE 1:**
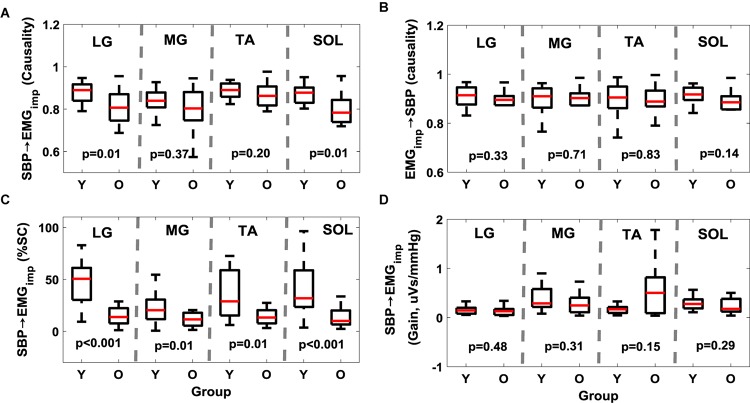
Effect of aging on muscle-pump baroreflex causality (**A**, SBP→EMG_imp_), muscle-pump (**B**, EMG_imp_→SBP), muscle-pump baroreflex %SC (**C**, SBP→EMG_imp_), and muscle-pump baroreflex gain (**D**, SBP→EMG_imp_) for four leg muscles. %SC and Gain in LF region are reported here. The figure lists the Wilcoxon rank sum comparison *p*-value between young (Y) and older (O) groups for respective leg muscles.

Further, while aging had no effect (*p* > 0.10) on the muscle-pump (EMG_imp_→SBP) for any muscles (Figure 1B and Table 3), the muscle-pump baroreflex of LG (*p* = 0.01) and SOL (*p* = 0.01) was significantly lower in the older group (Figure 1A and Table 3). The SBP→EMG_imp_ %SC was significantly lower in the older persons compared to young for all muscles (LG, *p* < 0.001; MG, *p* = 0.01; TA, *p* = 0.01; and SOL, *p* < 0.001) (Figure 1C and Table 3). No difference (*p* > 0.10) was observed in muscle-pump baroreflex (SBP→EMG_imp_) gain for any muscles between the young and older groups (Figure 1D and Table 3).

**TABLE 3:** Comparison (mean ± SD) of muscle-pump baroreflex causality, muscle-pump causality, muscle-pump baroreflex %SC, muscle-pump baroreflex gain (μV s/mmHg), cardiac baroreflex causality, cardiac baroreflex %SC, and cardiac baroreflex gain (ms/mmHg) between the two groups.

**Parameters**	**Young**	**Older**	***p*-Value**
SBP→EMG_imp (LG)_ causality	0.88 ± 0.05	0.81 ± 0.08	0.01^†^
SBP→EMG_imp (MG)_ causality	0.84 ± 0.06	0.80 ± 0.11	0.37
SBP→EMG_imp (TA)_ causality	0.89 ± 0.04	0.87 ± 0.05	0.20
SBP→EMG_imp (SOL)_ causality	0.88 ± 0.04	0.79 ± 0.11	0.01^†^
EMG_imp (LG)_ →SBP causality	0.91 ± 0.04	0.87 ± 0.09	0.33
EMG_imp (MG)_ →SBP causality	0.90 ± 0.05	0.87 ± 0.09	0.71
EMG_imp (TA)_ →SBP causality	0.90 ± 0.06	0.89 ± 0.06	0.83
EMG_imp (SOL)_ →SBP causality	0.91 ± 0.04	0.87 ± 0.08	0.14
SBP→RR	0.94 ± 0.03	0.88 ± 0.08	0.01^†^
RR→SBP	0.94 ± 0.03	0.92 ± 0.05	0.53
SBP→EMG_imp (LG)_ %SC	47.96 ± 23.86	14.63 ± 8.41	< 0.001^†^
SBP→EMG_imp (MG)_ %SC	23.86 ± 17.02	11.24 ± 6.61	0.01^†^
SBP→EMG_imp (TA)_ %SC	33.31 ± 22.64	16.56 ± 14.82	0.01^†^
SBP→EMG_imp (SOL)_ %SC	39.91 ± 24.80	12.90 ± 9.05	< 0.001^†^
SBP→RR %SC	76.75 ± 16.91	50.95 ± 23.31	0.001^†^
SBP→EMG_imp (LG)_ gain	0.15 ± 0.08	0.15 ± 0.13	0.48
SBP→EMG_imp (MG)_ gain	0.49 ± 0.52	0.35 ± 0.36	0.31
SBP→EMG_imp (TA)_ gain	0.31 ± 0.45	0.72 ± 0.81	0.15
SBP→EMG_imp (SOL)_ gain	0.28 ± 0.12	0.26 ± 0.21	0.29
SBP→RR gain	13.05 ± 8.11	4.39 ± 4.38	< 0.001^†^

*Gain and %SC in LF region are reported. Table also lists *p*-value for Wilcoxon rank sum test. ^†^Represents significant difference between the two groups.*

**FIGURE 2:**
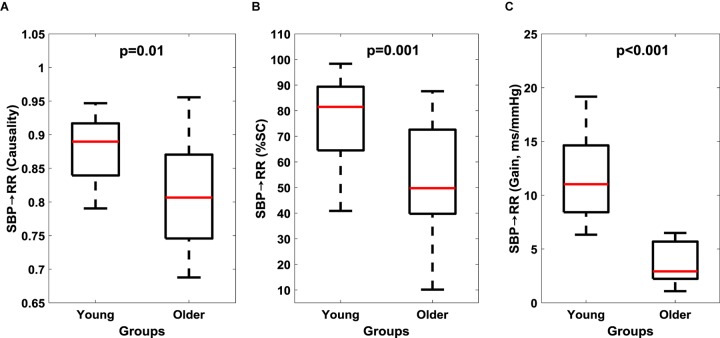
Comparison of cardiac baroreflex (SBP→RR) response between young and older groups. Baroreflex causality **(A)**, percent time with significant coherence **(B)**, and Gain **(C)** in LF region are reported here. The figure lists the Wilcoxon rank sum comparison *p*-value between young and older groups.

The non-linear interaction in the baroreflex direction (SBP→RR) was significantly lower (*p* = 0.01) in the older group compared to the young (Figure 2A and Table 3). The strength of feedforward interaction, i.e., RR→SBP did not differ in older persons compared to young (0.92 ± 0.05 vs. 0.94 ± 0.03, *p* = 0.53), while SBP→RR %SC (*p* = 0.001) and SBP→RR gain (*p* < 0.001) were lower in older persons (Figures 2B,C).

**FIGURE 3:**
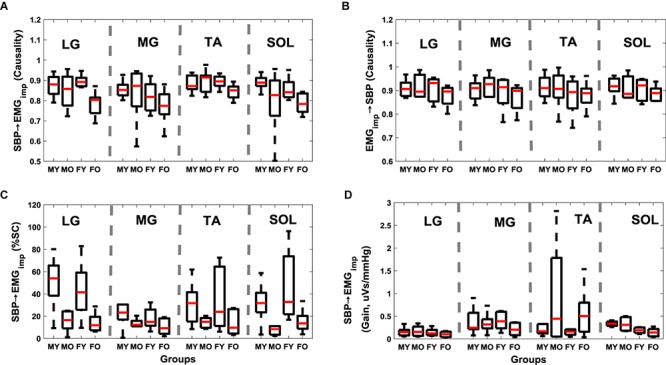
Distribution of muscle-pump baroreflex and muscle pump with age and sex for respective leg muscles. SBP→EMG_imp_ causality **(A)**, EMG_imp_→SBP causality **(B)**, SBP→EMG_imp_ %SC **(C)**, and SBP→EMG_imp_ gain **(D)**. Figure plots distribution of young males (MY), older males (MO), young females (FY), and older females (FO). SBP→EMG_imp_ %SC and SBP→EMG_imp_ gain in LF region are reported.

**FIGURE 4:**
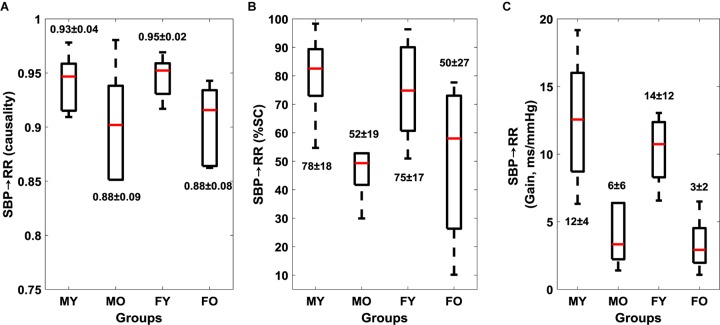
Effect of age and sex on cardiac baroreflex (mean ± SD). SBP→RR causality **(A)**, SBP→RR %SC **(B)**, and SBP→RR Gain **(C)**. Figure plots cardiac baroreflex for young males (MY), older males (MO), young females (FY), and older females (FO). SBP→RR %SC and SBP→RR gain in LF region are reported.

Muscle-pump baroreflex causality (SBP→EMG_imp_) of LG (*p* = 0.003), TA (0.02), and SOL (0.03) muscles were lower in older females compared to young females (Table 4). Lower SBP→EMG_imp_ %SC with age was observed for LG and SOL muscles, where both older males and females had lower value compared to their young counterparts (Table 4). A sex difference was observed in SBP→EMG_imp_ gain for SOL muscles, where young males had higher value compared to young females (*p* = 0.02) (Table 4 and Figure 3). An effect of aging on baroreflex causality was observed only in females where older females had a lower value compared to young females (Table 4 and Figure 4). Cardiac baroreflex gain was also lower in older females (*p* < 0.001) compared to their young counterparts (Table 4 and Figure 4).

**TABLE 4:** Comparative behavior of muscle-pump baroreflex and muscle-pump for different groups.

**Groups for comparison**	**MY vs. MO**	**MY vs. FY**	**MO vs. FO**	**FY vs. FO**
**Causality**				
SBP→EMG_imp (LG)_	0.79	0.63	0.22	0.003^†^
SBP→EMG_imp (MG)_	0.71	0.35	0.18	0.38
SBP→EMG_imp (TA)_	0.79	0.63	0.10	0.02
SBP→EMG_imp (SOL)_	0.42	0.10	0.66	0.03
SBP→RR	0.26	0.63	0.99	0.02
**%SC**				
SBP→EMG_imp (LG)_	0.003^†^	0.45	0.66	0.004^†^
SBP→EMG_imp (MG)_	0.05	0.40	0.85	0.16
SBP→EMG_imp (TA)_	0.05	0.82	0.66	0.19
SBP→EMG_imp (SOL)_	0.007^†^	0.76	0.34	0.02
SBP→RR	0.03	0.69	0.99	0.06
**Gain**				
SBP→EMG_imp (MG)_	0.99	0.82	0.34	0.50
SBP→EMG_imp (MG)_	0.99	0.51	0.41	0.19
SBP→EMG_imp (TA)_	0.71	0.82	0.85	0.13
SBP→EMG_imp (SOL)_	0.99	0.02	0.05	0.32
SBP→RR	0.03	0.57	0.66	< 0.001^†^

*The table list the comparison *p*-value for Wilcoxon rank sum test. After correction, results were considered significant at *p* ≤ 0.012 and 0.012<*p* ≤ 0.025 represents trend. ^†^Represents significant difference between the groups.*

## Discussion

Falls associated with orthostatic intolerance is a recognized cause of injury in older persons while conducting day-to-day activities. Assessment of orthostatic tolerance solely via autonomic control of blood pressure could be ineffective due to the influence of other physiological systems in the regulation of blood pressure during orthostatic challenge. Measurement of the postural system (leg muscle activation) in addition to autonomic control can provide supplemental insights regarding an individual’s ability to maintain blood pressure during orthostatic challenge. While leg muscles are known to pump venous blood back to the heart, the significant contribution of the reverse, i.e., activation of leg muscles in response to fluctuations in blood pressure (muscle-pump baroreflex) during standing was recently demonstrated by our laboratory (Xu et al., 2017). This article extends our previous work and quantified muscle-pump baroreflex of lateral gastrocnemius, medial gastrocnemius, tibialis anterior, and soleus muscles during standing. Further, we studied the effect of aging on muscle-pump baroreflex and its relationship with the cardiac baroreflex.

Aging is known to deteriorate cardiac baroreflex function, however, the effect of aging on muscle-pump baroreflex is not well understood. Poor muscle performance and impaired baroreflex control are accountable for postural instability and orthostatic hypotension; causing falls in older persons (Gazibara et al., 2014; Goswami, 2017). Therefore, comprehensive knowledge regarding aging-related changes in cardiac and muscle-pump baroreflexes can be consequential in the design of countermeasures to orthostatic intolerance. In this research, we exposed young and older individuals to orthostatic challenge induced via the stand test and compared the deviation in muscle-pump baroreflex for different leg muscles within and between the two age groups supplemental to cardiac baroreflex. In the young group, no change (*p* > 0.10) in EMG_imp_→SBP causality between the respective leg muscles was observed (Table 2). However, the muscle-pump baroreflex (SBP→EMG_imp_) for MG muscle was significantly lower compared to the TA (*p* = 0.04) muscle (Table 2), suggesting less reliance of MG muscles on baroreflex mediated activation compared to the TA. Furthermore, the strength of LG and SOL muscles on baroreflex mediated activation was similar to the TA (*p* = 0.98, *p* = 0.87, respectively). This observation revealed the reliance of LG, TA, and SOL muscles on baroreflex mediated activation during standing.

In the older group, the muscle-pump baroreflex was lower for LG (*p* = 0.01) and SOL (*p* = 0.01) muscles when compared to young group (Figure 1). While aging had no effect (*p* > 0.10) on the feedforward control of blood pressure (EMG_imp_→SBP) for any muscles, lower muscle-pump baroreflex symbolizes impairment in the baroreflex dependent activation of LG (*p* = 0.01) and SOL (*p* = 0.01) muscles toward pumping the venous blood back to the heart in the older group. Such deficiency, if not compensated via other regulatory mechanisms during orthostatic challenge, could lead to light-headedness resulting in an unexpected fall. In the young group, LG, TA, and SOL muscles were observed to be dependent on baroreflex mediated activation. Aging-related impairment in baroreflex dependent activation of LG and SOL muscles indicates reliance on TA as a compensatory mechanism to facilitate venous return to the heart, accordingly, higher (*p* = 0.001) EMG_imp_ for TA muscle in the older group was noticed compared to young. Moreover, %SC, signifying the percentage of time during standing with a significant interaction between SBP and respective leg muscles, was significantly lower for all muscles in older persons compared to young. This behavior emphasizes the aging-related muscle-pump baroreflex impairment through the reduction of the interaction (in terms of time) between blood pressure and leg muscles (for all muscles) during orthostatic challenge to maintain blood pressure homeostasis (Figure 1).

To examine the aging-related reduction in muscle-pump baroreflex causality (LG and SOL) and %SC (all muscles) with cardiac baroreceptor response; we compared the dynamics of cardiac baroreflex between the two groups. The baroreflex control quantified via baroreflex sensitivity (SBP→RR gain), causal heart period-blood pressure interaction (SBP→RR causality), and SBP→RR %SC was significantly lower in older group compared to young (Figure 2), highlighting the aging-related impairment of cardiac baroreflex. This finding was in alignment with literature where age-related impairment in baroreflex mediated heart rate control (SBP→RR) was observed (Porta et al., 2014). The observation of lower cardiac and muscle-pump baroreflexes in older compared to young persons underscored the importance of interaction between the cardiovascular and postural systems to facilitate blood pressure homeostasis, and when considered together could serve as a vital indicator of individual’s orthostatic tolerance.

Although, age-related impairment in muscle-pump baroreflex (SBP→EMG_imp_) was observed for LG and SOL muscles in terms of causality and all muscles in terms of %SC, the skeletal muscle activation (EMG_imp_) for respective muscles was not different, except for the TA, which was higher (*p* = 0.001) in the older compared to the young group. Studies in the literature have observed higher leg muscle activity in older persons compared to young (Laughton et al., 2003; Masterson and Morgan, 2006). Research has also indicated higher postural sway in older persons compared to young (Laughton et al., 2003; Roman-Liu, 2018). Further, higher postural sway has been shown to be associated with higher leg muscle activity in older persons (Laughton et al., 2003; Vette et al., 2017).

Our previous study, conducted on young participants, demonstrated significant closed-loop interaction between the summed muscle (LG+MG+TA+SOL) EMG_imp_ and postural sway during standing, where postural sway mediated muscle activation was observed to be higher than the reverse (Xu et al., 2017). This underscored the interplay of postural sway mediated activation leg muscles and the muscle-pump baroreflex in the maintenance of posture and blood pressure. Although postural muscle contractions will produce transient increases in venous return, these may not occur at a nadir in blood pressure and have minimal effect on blood pressure regulation. A baroreceptor reflex mediated coordination of the muscle-pump with blood pressure can provide an efficient response to orthostatic hypotension when needed even in otherwise healthy young individuals. Accordingly, in the current study, no difference (*p* > 0.10) in muscle-pump baroreflex gain was observed in older persons compared to young (Figure 1D) suggesting that the strength of the relationship was unaltered with age in healthy, active adults in spite of the reduction in causality and %SC with aging.

Research into syncope in humans has shown that females have lower orthostatic tolerance compared to their male counterparts (White et al., 1996; Convertino, 1998; Shoemaker et al., 2001). This sex effect has been observed in the cardio-postural blood pressure control during standing (Xu et al., 2017). Here, we observed lower SBP→RR causality (*p* = 0.02) in older females compared to their young counterparts, similarly, SBP→RR causality in older males was lower compared to young males but did not achieve statistical significance (Figure 4 and Table 4). Furthermore, we observed lower muscle-pump baroreflex causality in older females compared to young females for LG (*p* = 0.003), TA (*p* = 0.02), while no difference (*p* > 0.10) was observed in older males compared to young males (Table 4). These observations suggest that older females may be more vulnerable to orthostatic intolerance compared to older males due to impairment in muscle-pump baroreflex of LG and SOL muscles. However, further investigation by utilizing a bigger sample size is required to generalize the findings of the current study.

The findings of the study are promising and could aid in early identification of orthostatic intolerance by continuous monitoring of cardiac and muscle-pump baroreflexes. Traditionally, assessment of orthostatic intolerance is based on cardiac baroreflex, incorporation of muscle-pump baroreflex could further improve the reliability of a system designed for monitoring an individual’s orthostatic tolerance to mitigate potential associated fall. Further, the finding of the study can assist in the design of specific exercise or training strategies to negate aging-related impairment in muscle-pump baroreflexes.

## Conclusion, Limitation, and Future Direction

This article extended our previous work and studied the effect of aging on muscle-pump baroreflex of LG, MG, TA, and SOL muscles in addition to cardiac baroreflex. We observed lower cardiac baroreflex control of blood pressure in the older group compared to young (Figure 2). Similarly, muscle-pump baroreflex causality of LG and SOL muscles and muscle-pump baroreflex %SC for all muscles were lower in the older group compared to young (Figure 1). No change (*p* > 0.10) in mechanical muscle pump (EMG_imp_→SBP) of any muscle was observed, which highlights aging-associated impairment only in the baroreflex-mediated control of leg muscle activation (LG and SOL muscles). Additionally, age related change in females was observed for cardiac and muscle-pump baroreflexes, where females showed lower values compared to males with age (Table 4), which suggests older females could be more vulnerable to orthostatic intolerance compared to older males. In a nutshell, the findings of the current study exhibited the degree of dependency of respective leg muscles on baroreflex-mediated activation and further impairment in the same function with aging.

The limitation of the present study was the unavailability of the center of pressure data, as such, the role of postural sway in the activation of respective leg muscles couldn’t be verified. As a result, further study is warranted to differentiate baroreflex and posture mediated activation of leg muscles. Moreover, near-infrared spectroscopy can be utilized in a future study to accurately measure the degree of blood pooling in the calf during standing (Blaber et al., 2013a), such data will further improve our physiological understanding of the relationship between blood pooling in the legs and muscle-pump baroreflex of lower leg muscles. Moreover, this study did not measure respiration which is shown to mediate changes both in blood pressure and heart period (Porta et al., 2012), therefore, future studies should also incorporate respiration when addressing physiology associated with blood pressure regulation. Additionally, the model considered in this study for quantifying physiological interaction was bivariate, incorporating center of pressure and respiration in future research would demand multi-variate model for the analyses of physiological interaction (Porta et al., 2012; Wen et al., 2013; Porta and Faes, 2016). As well, the sample size of each group considered for the analysis was limited, accordingly, future analysis with bigger cohorts is required to comprehensively validate the effect of sex and aging on muscle-pump baroreflex during orthostatic challenge. Finally, it should be noted that the older individuals who participated in this study were healthy and physically active, accordingly, they were able to negate the effect of impairment in cardiac and muscle-pump baroreflexes via higher activation of TA muscle, as such orthostatic hypotension was not experienced by older participants during standing. Extension of this analysis to a population with a history of orthostatic hypotension, where activating leg muscle may be challenging, is warranted to validate the promise of cardio-postural interaction for assessment of orthostatic tolerance.

## Data Availability

The datasets generated for this study are available on request to the corresponding author.

## Ethics Statement

The experimental design was approved to be of minimal risk by the research ethics board of Simon Fraser University (SFU). Written and informed consent for participation was obtained from all participants prior to experimentation.

## Author Contributions

AV, AG, AB, and KT conceived the research. AB conceived the cardio-postural model. AG and AB recorded the data. DX preprocessed the data. AV, DX, and AG developed the analysis methodologies. AV conducted the data and statistical analysis and wrote the manuscript. All authors critically edited the manuscript and approved the final version for submission.

## Conflict of Interest Statement

The authors declare that the research was conducted in the absence of any commercial or financial relationships that could be construed as a potential conflict of interest.
